# Managing tuberculosis infection among migrants from high-incidence tuberculosis countries: challenges, strategies and recommendations

**DOI:** 10.1007/s10096-025-05301-7

**Published:** 2025-10-09

**Authors:** Salvatore Rotundo, Francesca Serapide, Gabriella d’Ettorre, Maria Teresa Tassone, Mattia Albanese, Gabriella Giuseppina Marino, Bruno Tassone, Giancarlo Ceccarelli, Alessandro Russo

**Affiliations:** 1 Department of Medical and Surgical Sciences, “Magna Graecia” University, Catanzaro, Italy; 2 Infectious and Tropical Disease Unit, “Renato Dulbecco” Teaching Hospital, Catanzaro, Italy; 3 Department of Public Health and Infectious Diseases, University “Sapienza” of Rome, Rome, Italy

**Keywords:** Tuberculosis, migrants, BCG, screening, diagnosis, IGRA, TST, treatment, rifapentine, adherence

## Abstract

Tuberculosis (TB) remains a significant global health challenge, especially in countries with low TB incidence, exacerbated by the influx of migrants from high TB-burden regions. This paper reviews the challenges and strategies for managing TB infection (TBI) among migrants. Challenges in screening and treating TBI among migrants include diagnostic limitations of available tests which are tuberculin skin test (TST) and interferon-gamma release assay (IGRA), socioeconomic barriers, cultural beliefs and mobility. Recommendations vary among guidelines, ranging from proactive screening to targeted approaches. We addressed the issue of two-step testing, discussing the use of an initial TST followed by IGRA confirmation, with consideration of BCG vaccination status and TB exposure history. Treatment options for TBI include isoniazid monotherapy and rifamycin-based regimens in most cases, with varying preferences across guidelines. Challenges in TBI treatment include hepatotoxicity and adherence issues, particularly among migrants. Overall, a comprehensive approach addressing socioeconomic, cultural, and structural factors is crucial for effective TBI management among migrants. Collaboration between healthcare providers, policymakers and migrant communities is essential for developing culturally sensitive screening and treatment protocols. Further research is needed to evaluate the efficacy and feasibility of different screening and treatment strategies, particularly among migrant populations.

## Introduction

Tuberculosis (TB) remains one of the most pressing global public health challenges. Despite the World Health Organization (WHO) setting an ambitious goal to reduce TB-related deaths by 95% by 2030, significant disparities persist, driven by different factors including social determinants, gender inequities, migration dynamics, and recent geopolitical changes [1–5]. This goal has been further hindered by the COVID-19 pandemic, which diverted healthcare resources away from TB control efforts, likely contributing to a resurgence of the disease globally. Indeed, as the pandemic wanes, TB has re-emerged as the leading cause of death attributable to a single infectious agent [6]. 

According to the 2024 Global Tuberculosis Report by the WHO, approximately 10.8 million people developed TB in 2023, with over 1.25 million deaths attributed to the disease [6]. However, these numbers reflect not only the burden of a pathogen but also the deep-rooted social and economic inequalities that drive its spread across different continents. Indeed, TB epidemiology clearly shows that the disease disproportionately affects low- and middle-income countries, where 98% of TB-related deaths occur. Southeast Asia and Sub-Saharan Africa bear the brunt of the burden, contributing 45% and 24% of global cases, respectively [6]. In some migrants’ countries of origins TB remains a severe public health emergency, in particular 30 high-TB-incidence countries account for 87% of global cases, with India, China, Indonesia, Nigeria, Pakistan, and South Africa leading in the absolute number of new cases [6]. Indeed, TB is closely linked to poverty and marginalization, disproportionately affecting vulnerable communities [3]. This is driven by a complex interplay of social determinants, including undernutrition, overcrowded living conditions, unsafe working environments, limited access to healthcare, social stigma associated with TB, and comorbidities such as HIV coinfection. These factors often act synergistically, compounding the disease burden in already marginalized populations [6–11]. Addressing TB in these countries is further complicated by the need to balance emergency interventions for active TB with prevention programs for TB infection (TBI) [12]. In many high-incidence TB countries, TBI prevalence rates exceed 40%, yet preventive treatment programs are often restricted to specific groups, such as healthcare workers [13]. Indeed, in such settings, healthcare resources are scarce and primarily dedicated to diagnosing and treating active TB rather than TBI [14].

**Fig. 1:**
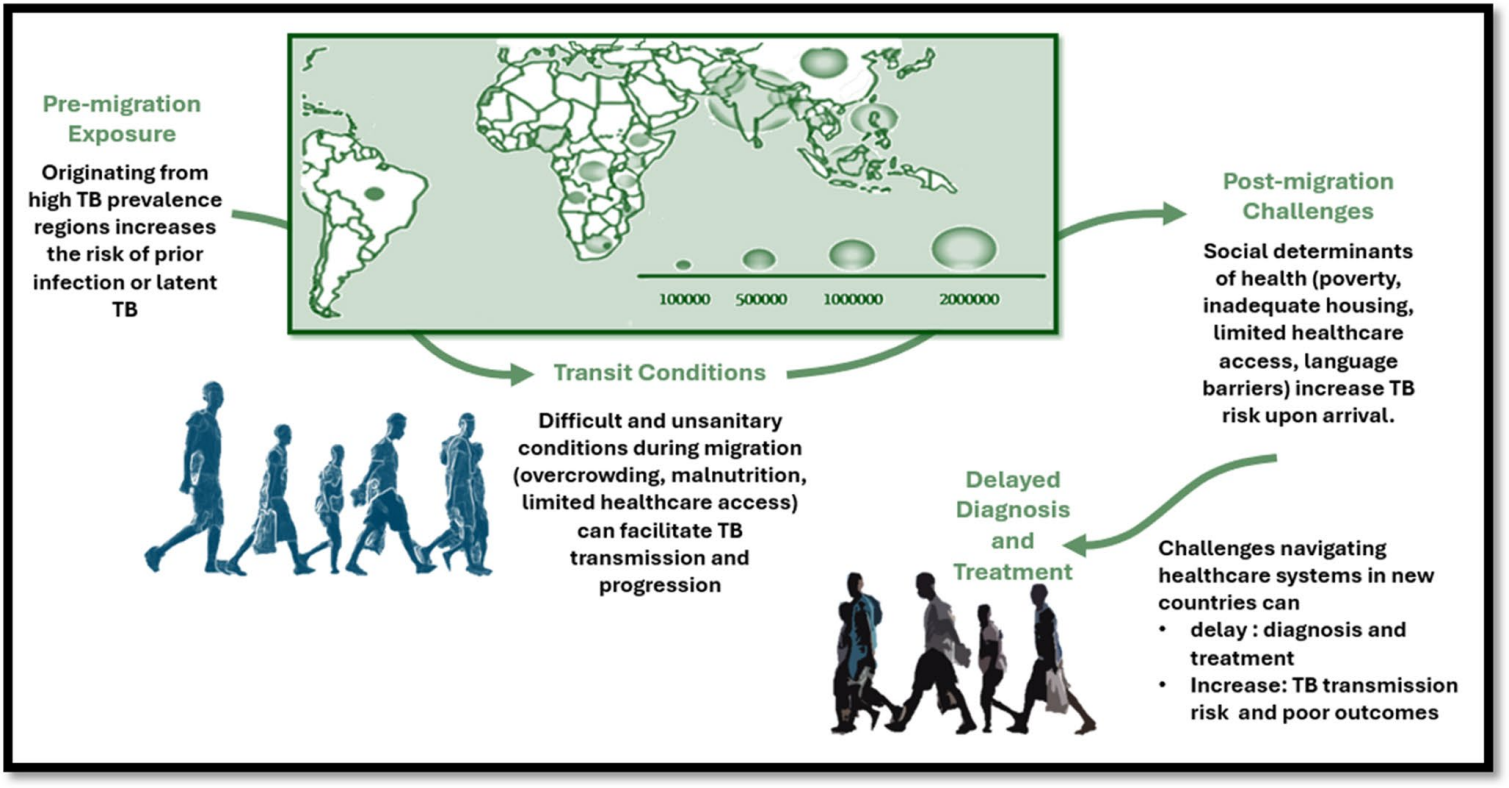
Factors contributing to the elevated risk of Mycobacterium tuberculosis infection and disease among migrants, especially those from high-burden countries. Global map displaying estimated incident tuberculosis case counts for nations reporting 100,000 or more cases in 2022 (source: adapted from WHO Global Tuberculosis Report 2023 [6])

Even in countries with low TB incidence, despite reactivations in elderly individuals previously infected, clusters in daycare centers and communities, and the heightened risk among the immunocompromised due to chronic conditions or iatrogenic causes, this disease remains closely linked to poverty and marginalization [15–17]. It disproportionately affects vulnerable individuals such as migrants, further exacerbating pre-existing inequalities [3]. As of recent data, approximately 13% of the European region's total population were foreign-born with the Russia–Ukraine conflict laeding to a substantial increase in the number of refugees seeking shelter in neighboring countries, crossing borders in search of safety and security [18]. Grapplingwith this disease is further complicated by the ongoing migration crisis that underscores the need for multifaceted approaches to healthcare delivery and public health policy [2]. For instance, in Italy, the TB epidemiological landscape mirrors global dynamics with some local differences [2, 5, 19–21]. Significant socioeconomic disparities are evident even within the country, with southern and insular regions often showing poorer favorable indicators compared to northern areas [22]. The recent increase in migration flows, predominantly affecting southern regions and islands, has introduced additional challenges in managing TB [2, 5, 23]. Intervening to treat TBI before it evolves into active disease can effectively halt onward transmission and mitigate the associated risks and costs of active TB [24]. Consequently, effective management of TBI among migrants emerges as a pivotal strategy in the pursuit of TB elimination goals [25] (Fig. 1). 

Further complicating the global landscape, the COVID-19 pandemic deeply disrupted healthcare systems, diverting critical resources and attention from TB control efforts. This disruption not only delayed diagnoses and treatments but also led to an increase in the number of people living in poverty, particularly in low income countries, where the compounded effects of economic instability and weakened healthcare infrastructure heightened vulnerability to both TB and other infectious diseases [26]. Lockdowns and restrictive measures likely hindered TB screening, diagnostics, and follow-up activities, potentially causing delays in diagnosis and treatment. This, in turn, may have increased the risk of further disease transmission as the pandemic came under control [20, 27, 28]. Moreover, the economic crisis triggered by the pandemic significantly reduced healthcare funding, further deepening existing disparities in access to TB care [26, 29]. Despite various mitigation strategies to manage the pandemic, these interventions proved insufficient to protect socioeconomically vulnerable populations adequately [30]. Many patients, particularly those from disadvantaged backgrounds, faced economic and practical barriers to accessing treatment, including transportation difficulties and the affordability of medications during the pandemic [26, 29]. Marginalized groups, such as migrant workers and refugees, were largely overlooked in formal policy responses [30]. Furthermore, the inability to adopt preventive measures like social distancing, mask use, and proper hygiene exacerbated the spread of TB and COVID-19. Many vulnerable populations rely on informal or unregulated work, making it impossible to adhere to distancing measures without sufficient economic support [26].

## Challenges for care migrants with tuberculosis infection

Assessing the true prevalence of TBI in migrants is challenging due to the absence of a direct diagnostic test and the non-mandatory reporting in most low-incidence countries [31]. Despite these challenges, it was suggested that TBI rates among migrants from high-incidence countries can exceed 40% [32]. Moreover, it’s important to note that post-entry screening has revealed a higher prevalence of TB among migrants compared to the estimated prevalence in their countries of origin. This highlights the potential impact of travel conditions and duration on the risk of developing active TB. Therefore, when considering whether to recommend screening for TBI to a migrant, their travel history should be taken into account alongside other relevant risk factors [19]. A recent report on a cluster of Eritrean migrants rescued from a Mediterranean shipwreck is particularly relevant in this context [33]. The report revealed widespread undernutrition, including deficiencies in cobalamin, folic acid, and notably, vitamin D. This vulnerability appeared correlated with reported poor living conditions during their journey and prolonged detention in Libya. Exacerbating this situation is the inherent vulnerability of Sub-Saharan African refugees to vitamin D deficiency and linked to a history of limited sunlight exposure in refugee camps and Libyan detention centers [34–37]. Furthermore, vitamin D deficiency and undernutrition are closely linked to poor overall health. This is further connected to the crucial role of vitamin D in immune function and the increased risk of infections, including pneumonia and TB, associated with its deficiency [38–40].

As migrants move across borders, they may bring with them health needs, including TBI screening, treatment and prevention since there is a considerable burden of TBI in this population which needs robust surveillance and intervention strategies to address this public health concern effectively. However, migrants encounter numerous obstacles in accessing and adhering to TBI treatment, stemming from various socioeconomic, cultural, and structural factors and may have unique TB risk factors and exposure histories, necessitating tailored TBI screening protocols followed by appropriate treatment strategies [19]. Language barriers often hinder migrants’ comprehension of healthcare information and impede effective communication with healthcare providers [41]. Consequently, accessing the healthcare system becomes challenging, leading to misunderstandings about TBI treatment and difficulties in following treatment instructions [42]. Financial constraints exacerbate the situation, particularly for migrants with undocumented status or limited resources. Financial limitations may prevent them from affording transportation to healthcare facilities, purchasing medications or taking time off work for medical appointments [43]. Moreover, migrants frequently face significant socioeconomic obstacles, including poverty, unstable housing, and unemployment. These challenges not only hinder their access to and compliance with TBI screening but also increase their risk to developing active TB. Additionally, the complex socioeconomic factors intertwined with migration, such as overcrowded living conditions and limited healthcare access, can amplify TB transmission and impede the implementation of effective control measures [44]. Cultural and religious beliefs significantly influence migrants’ perceptions of health and illness, shaping their attitudes towards medical treatment. Some migrants may hold beliefs conflicting with Western medical practices or harbor concerns about the safety and efficacy of TBI treatment, resulting in treatment refusal or non-adherence [45]. Stigma and discrimination related to TB complicate the issue, both within migrant communities and the broader society. Fear of being labeled as TB carriers or facing social ostracism discourages migrants from seeking TBI testing and treatment or disclosing their TB status [46]. The transient nature of migration poses another challenge, with migrants frequently moving between locations due to work, housing, or family reasons. This mobility may disrupt TBI treatment continuity, leading to interruptions in medication adherence and follow-up care [47]. Furthermore, migrants often lack knowledge or awareness about TB, including its transmission, prevention, and treatment. Limited knowledge about TBI and its significance contributes to low treatment initiation rates and poor treatment outcomes among migrants [48]. Indeed, while migrants from high TB-prevalence countries are typically familiar with active TB disease, they are often less aware of TBI. As a result, when informed they have TBI, they may mistake it for active disease, leading to unnecessary anxiety [49].

Addressing these multifaceted challenges necessitates a comprehensive approach that tackles the social, economic, cultural and structural determinants of health among migrant populations. Initiatives to improve TBI treatment access and adherence among migrants should encompass culturally and linguistically appropriate healthcare services, community-based outreach and education programs, and policies addressing social and economic barriers to healthcare access. Collaboration between healthcare providers, migrant communities, and policymakers is crucial for developing and implementing effective strategies for TBI prevention and care in migrant populations. Recognizing the diverse cultural backgrounds and beliefs of migrant populations is essential for providing culturally competent care. Healthcare providers should be trained to understand and respect cultural differences, ensuring effective communication and collaboration with migrant patients.

## Screening for tuberculosis infection among migrants

Managing TBI testing among migrants presents several challenges. These include, among others, the presence of trained personnel, laboratory capabilities, and financial resources necessary to fund testing.

Symptomatic/syndromic surveillance for TB often serves as the initial step in screening newly arrived migrants [50]. This approach involves actively inquiring about the presence of TB-related symptoms, such as cough, fever, night sweats, and weight loss [51]. While practical as a first step, particularly in out-of-hospital setting, this method has limitations. Not all individuals with TB exhibit symptoms, especially in the early stages of the disease or in cases of extrapulmonary TB. Moreover, symptoms like cough and fever are common to many other respiratory illnesses, potentially leading to false positives and unnecessary further investigations [52–54]. Despite its limitations, symptom-based screening can be a valuable tool for identifying individuals who warrant further evaluation for TB, particularly in high-risk populations like migrants from TB endemic settings. While syndromic evaluation remains the prevailing approach for initial migrant health assessments, its limitations warrant attention. Migrants, even upon arrival without overt signs of disease such as TB, may already exhibit a decline in health status attributable to the combined stressors of extended travel and prolonged detention. This phenomenon, potentially indicative of a premature “exhausted migrant effect”, can precipitate the early onset of frailty-related morbidities, posing a significant threat to both individual and public health [33].

Currently, there is not a universally accepted reference method for diagnosing TBI [24, 41, 55] and despite both tuberculin skin test (TST) and interferon-gamma releasing assay (IGRA) as valuable tools for this purpose, they have limitations that should be considered in the decision to test migrants from high-incidence countries [56]. A summary of the comparative characteristics of these tests is provided in Table 1. 

**Table 1:** Comparison of characteristics between tuberculin skin test (TST) and Interferon-Gamma releasing assay (IGRA). ESAT-6: early secretory antigenic Target-6; CFP-10: culture filtrate Protein-10; BCG: Bacillus Calmette-Guérin; NTM: Non-tuberculous mycobacteria; MTB: Mycobacterium tuberculosis complex

Characteristics	Tuberculin skin test	Interferon-gamma releasing assay
Type of test	In vivo	In vitro
Antigen	Purified protein derivative	*Mycobacterium tuberculosis*-specific antigens (ESAT-6, CFP-10)
Specificity	Cross-reactivity with BCG vaccination and NTM	Not affected by BCG vaccination and only to a few NTM (i.e., *M. kansaasi*, *M. szulgai*, *M. marinum*)
Administration	Intradermal injection. Requires cold chain for storage.	Requires blood draw
Interpretation	Subjective interpretation based on induration size	More specific measurement of interferon-gamma release production against MTB antigens
Turnaround time	Second visit required for interpretation (48–72 h)	Results typically available within 8–30 h
Cost	Lower	Higher
Applicability	Commonly used in resource-limited settings	Suitable for children older than two years, especially in those with previous BCG vaccination

Importantly, the existing tests cannot distinguish between present and past infection, as they detect immunological memory to *Mycobacterium tuberculosis* complex antigens but cannot determine when the infection occurred. Therefore, another limitation of these tests stem from their inability to discern between cleared infection, ongoing subclinical infection and incipient/asymptomatic or active TB disease [56]. Moreover, both TST and IGRA have technical limitations. Regarding TST, optimal accuracy in both administering the test and interpreting the resulting skin reaction is important. For reliable test results, a TST requires administration and interpretation by a healthcare provider who has undergone specialized training in the procedure [57]. Conducting IGRA procedure relies not only on the expertise of trained professionals but also on having the necessary laboratory facilities and costly instrumentation [55]. IGRA necessitates a phlebotomy procedure, which may not be acceptable for some migrants. Indeed, in certain cultural contexts, there is significant reluctance to undergo blood drawing with could be a limitation in wide use of IGRA tests in migrant populations. This hesitance is often driven by deep-seated beliefs linking blood loss to vulnerability and superstition. For instance, in many African societies, individuals fear potential consequences such as being cursed or falling victim to black magic as a result of blood sampling [58]. In addition, IGRA testing can sometimes yield indeterminate or borderline results, which adds further complexity to the screening process. These inconclusive outcomes can arise due to various factors, including immune suppression, technical variability, or improper sample handling [59, 60]. Consequently, results near the diagnostic threshold are prone to variability and may require retesting or clinical judgment for interpretation [60]. In routine screening settings can significantly impact clinical decision-making, especially in low-endemicity contexts where the pre-test probability of infection is low [59]. However, IGRA presents the advantage of yielding results in just one visit, whereas TST necessitates a second visit to interpret the test outcome [55]. Lastly, IGRA typically offers faster results compared to the TST. While the TST requires interpretation 48 to 72 h after administration, IGRA results can typically be obtained within 8 to 30 h after the blood sample is collected [31]. Additionally, both TST and IGRA exhibit limited predictive power for the progression to active TB once they yield positive results [25, 61]. Although both IGRA and TST are considered to be highly specific [31], TST is more likely to produce false positive results [24, 62]. It is noteworthy that non-tuberculous mycobacteria (NTM) play a limited role in causing false-positive TST results, except in populations characterized by a high prevalence of NTM sensitization and a very low prevalence of TBI. In such contexts, the presence of NTM may contribute to false-positive TST reactions, necessitating careful consideration of local epidemiological factors when interpreting TST results [63]. A false-positive TST result can occur more frequently due to cross-reactivity in individuals who have received the Bacillus Calmette-Guérin (BCG) vaccine [3] which is still widely administered in high-incidence TB countries since play a pivotal role in preventing childhood TB meningitis and miliary disease [64] and, despite the impact of BCG vaccination received in infancy on TST results is generally minimal, particularly when assessed at least 10 years from vaccination, BCG vaccination administered after infancy often leads to more frequent, persistent, and larger TST reactions [63]. Despite these limitations, in contrast to other international guidelines, NICE guidelines prioritizes sensitivity over specificity for recent arrivals from high-TB-incidence countries. Therefore, TST is recommended as the initial test, with a cutoff of 5 mm [24]. Moreover, TST offers advantages even in children because the immaturity of their immune system makes them more likely to test negative or not being able to respond functionally with IGRA [24].

Recommendations across international guidelines [24, 31, 41, 55, 57, 61, 65] are shaped by disparities in epidemiological landscapes, healthcare systems, and resource availability among nations, as reflected in the overview provided in Table 2, which highlights the diverse approaches adopted globally.

**Table 2:** Screening recommendations for migrants from high-incidence tuberculosis (TB) areas according to international guidelines. IGRA: interferon gamma release Assay; TST: tuberculin skin Test; BCG: Bacillus of Calmette-Guérin

Organization, last update (year)	Preferred test	Key considerations
World Health Organization, 2025 [55]	IGRA or TST	In most countries globally, BCG vaccination administered at birth has a limited impact on TST specificity
National Institute for Health and Care Excellence, 2024 [24]	TST	More sensitive, accessible, cost-effective and familiar among healthcare providers
United States Preventive Services Task Force, 2023 [31]	IGRA or TST	Both TST and IGRA exhibit high specificity
National Tuberculosis Advisory Committee, 2022 [65]	IGRA	IGRA could potentially be the optimal choice for screening in children who have previously received the BCG vaccine
Canadian Tuberculosis Standards, 2022 [61]	IGRA or TST	Routine mass screening of all migrants is discouraged
Centers for Disease Control and Prevention, 2021 [57]	IGRA	Higher specificity, preferred over TST in people who received BCG vaccine, especially in low TB incidence settings
European Centre for Disease Prevention and Control, 2018 [41]	IGRA	IGRA eliminates the need for a follow-up visit to interpret the result, whereas TST accuracy can be impacted by prior BCG vaccination

The Centers for Disease Control and Prevention (CDC) guidelines recommends testing all migrants from high TB-endemic countries who have received the BCG vaccine with the IGRA. However, for children under two years old, regardless of BCG vaccination status, the preferred screening method is the TST [57]. This recommendation is due to some experts prioritizing sensitivity of the diagnostic test over its specificity due to the heightened susceptibility of young children to progressing to active TB. The urgency for early detection arises from the serious consequences of delayed treatment, which outweigh the reduced risk of hepatotoxicity due to antituberculosis drugs in this age group [66]. Additionally, IGRA are more likely to yield indeterminate results in children under two years of age [67].

Australian guidelines from the National Tuberculosis Advisory Committee (NTAC) prioritize recent migrants with TB contact within the last 24 months or those from high-incidence countries, particularly individuals under 35 or over 35 years with additional risk factors for TB progression. Both the TST and IGRA are acceptable options for screening in these cases [65]. However, for children aged two to ten years arriving from high TB incidence areas, NTAC recommends prioritizing IGRA testing over TST. This approach aims to minimize the likelihood of obtaining false positive results in children who have recently received the BCG vaccination [68].

The World Health Organization (WHO) stresses the need to screen migrants from high TB burden countries within five years of their arrival, but they do not endorse any specific tests for this purpose. Indeed, these guidelines suggest to not consider BCG vaccination status as a primary determinant when selecting a screening test for TBI [55]. However, it’s important to note that the effect of BCG vaccination on the specificity of TST results can vary depending on several factors, including the strain of the vaccine, the age at which it’s administered and the number of doses given [55]. For instance, TST specificity for TBI might be compromised in countries where revaccination with BCG or recent cessation of revaccination programs is common [69]. In a large Chinese cohort, Gao et al. [70] observed a moderate agreement between TST and IGRA. Notably, individuals who tested positive only on the TST were more likely to have a BCG scar, be male and be aged 60 years or older. Conversely, those who tested positive only on the IGRA were more likely to be male and aged 60 years or older. These findings suggest that the presence of a BCG vaccine scar could significantly contribute to the differences observed in the results of the two tests. In such contexts, IGRA may exhibit greater specificity compared to TST. Conversely, TST should not encounter issues with non-specificity in countries where BCG vaccination is administered once at birth [69].

According to the WHO, the European guidelines by both the European Centre for Disease Prevention and Control (ECDC) [41] and the National Institute for Health and Care Excellence (NICE) [24] advocate for a proactive approach to TBI screening among migrants. Indeed, according to NICE guidelines, it is recommended to conduct testing and provide treatment for everyone newly arriving from regions with a high prevalence of TB and seeking healthcare services. Particular attention should be paid for vulnerable migrants, a diverse group that encompasses individuals such as undocumented migrants and those facing barriers to accessing public funds. This category also includes refugees, asylum seekers, and individuals who have recently arrived in the country and may face unique challenges in accessing healthcare services [24]. Screening methods recommended by the ECDC include both TST and IGRA. Furthermore, these guidelines emphasize the importance of not only identifying TBI but also ensuring seamless linkage to appropriate care and treatment services for individuals identified through screening [71, 72]. By contrast Canadian Tuberculosis Standards (CTS) [61] discourage screening for TBI in all migrants since only certain subgroups of migrants facing risk factors, such as homelessness, substance abuse or incarceration, may require targeted TBI screening approaches [61]. According to CTS, achieving TB elimination (i.e., annual incidence of at most one case per million) in low-incidence countries will necessitate comprehensive screening and treatment of TBI. This includes individuals for whom the potential harms of TBI treatment may outweigh the expected benefits, such as older foreign-born individuals with no recent travel or exposure to TB raising a significant ethical and practical concern in TB control strategies [61]. Indeed, the low specificity of TBI diagnostics means that a substantial proportion of individuals who test positive may never develop active TB. Consequently, administering TBI treatment to these individuals, especially in low-incidence countries, could lead to overtreatment and unnecessary exposure to potentially harmful medications [73].

The United States Preventive Services Task Force (USPSTF) recently updated its 2016 recommendations [31], underlining the importance of ensuring proper treatment for individuals who test positive for TBI using either the TST or IGRA to prevent progression to active TB disease. USPSTF assumes high specificity for both TST and IGRA in detecting TBI, based on a systematic review revealed TST specificity ranging from 95% to 99%, primarily in studies conducted in low-incidence countries without BCG vaccination [74]. However, this review lacks studies involving a significant portion of BCG-vaccinated people. The sole study by Kastenos et al. [33] involving BCG-vaccinated individuals showed discrepancies between TST and IGRA results, suggesting that post-infancy BCG vaccination may significantly affect TST reactions [75]. Additionally, a meta-analysis covering 50,592 individuals across 41 cohorts found that IGRA had superior predictive accuracy for TB progression compared to TST. This indicates a higher risk of TB development among IGRA-positive individuals, particularly within migrant populations, despite both tests showing comparable sensitivity and specificity for prevalent TB [76].

In summary, guidelines vary on screening approaches, with some advocating for proactive screening of all migrants from high TB burden countries, while others recommend targeted screening based on specific risk factors. The interpretation of both IGRA and TST results in migrants can be complex, requiring consideration of factors such as prior BCG vaccination for TST and recent TB exposure in conjunction with clinical and epidemiological factors, that are imperative for precise diagnosis and effective management of TBI in such at risk population.

### A two-step approach for testing migrants

In a two-step testing approach, an individual receives TST and then IGRA within a short time frame, since a positive TST can interfere with IGRA results [6]. The first test serves as a baseline, and the second test is performed to confirm the initial result. It is essential that the two tests are performed relatively close together. Performing the tests too far apart could result in a loss of the boosting effect, leading to inaccurate interpretation of subsequent test results; conversely, performing an IGRA too soon after a TST may induce a boosting of interferon-gamma responses, potentially leading to false-positive results [77]. Additionally, delaying the second test increases the risk of potential TB exposure between tests, which could impact results. Although TST should not encounter issues with non-specificity in countries where BCG vaccination is administered only at birth [69], the evidence suggests that post-infancy BCG vaccination can significantly affect TST results. Indeed, discrepancies between TST and IGRA in BCG-vaccinated individuals are not uncommon, indicating that prior vaccination beyond infancy may lead to false-positive TST reactions [75]. Furthermore, IGRA has superior predictive accuracy for progression to active TB compared to TST, particularly among migrant populations [76]. Taken together, these findings support the recommendation that, in populations with high rates of BCG vaccination—such as migrants from high TB-burden countries—confirmatory testing with IGRA may be advisable to improve diagnostic precision and better identify individuals at genuine risk of developing TB. In particular, when TST results are uncertain, IGRA can serve as a valuable sequential test to enhance specificity [55, 78].

Despite a strategy involving a one test approach being more effective in general population [79], implementing sequential screening, particularly in BCG-vaccinated populations, could be the most cost-effective approach [80]. Implementing a sequential strategy for TBI screening with both TST and IGRA among migrants may indeed be justified by its higher cost-effectiveness, despite the lower completion rates of the screening cascade. However, IGRA-only strategy appears to have a higher likelihood of completing the screening cascade compared to those assigned to the sequential strategy [81, 82].

Assessing the cost-effectiveness of TBI screening among migrants involves three primary considerations. Firstly, the intervention’s standalone cost-effectiveness is presumed, aligning with WHO guidelines recommending systematic TBI testing and treatment for immigrants from high-TB-burden countries [55]. Secondly, evaluating which screening strategy (IGRA, TST or sequential TST/IGRA) is most cost-effective reveals sequential screening as the favorable option from both healthcare and economic standpoints. Test accuracy, particularly influenced by factors like BCG vaccination, underscores the importance of sequential testing. Thirdly, examining the cost-effectiveness of various testing strategies among migrants underscores the superiority of the sequential strategy over IGRA-only, consistent with findings from other studies [25], especially in BCG-vaccinated migrants form high-incidence TB countries [83]. Initial economic assessments indicate that targeting young migrants from countries with high TB incidence may represent the most cost-effective strategy [84]. However, further comprehensive economic analyses are warranted to confirm and refine these findings [25]. Interestingly, individuals testing positive for both IGRA and TST showed a higher risk of TB progression rather those who tested positive to only one of these tests [76], while it seems to be more cost effective compared with single-testing alone [79, 85, 86]. Indeed, the slightly lower specificity of the TST compared with IGRA could lead to an overestimation of the individuals who need TBI treatment, consequently inflating healthcare costs [87]. Similarly, while IGRA may offer the advantage of improved specificity in BCG vaccinated populations, widespread utilization of IGRA as a screening test presents its own economic challenges even for high-income countries [24, 55].

Therefore, the feasibility of the two-step approach remains a subject of debate. While some experts view it as more effective from both an economic and diagnostic standpoint, there are concerns about the low completion rates of the screening process.

## Treatment in migrants with tuberculosis infection

The primary objectives of treating individuals with TBI are twofold. On an individual level, the treatment aims to eliminate any dormant bacilli to prevent the later development of active TB [24]. From a public health perspective, treating TBI helps reduce the overall burden of TB within populations. This not only contributes to TB control and elimination initiatives within communities [41] but it has also been suggested that TBI treatment could prevent the emergence of drug-resistant strains [88]. However, the decision to proceed with treatment must carefully consider the individual patient’s risk of progressing to active TB weighed against the potential for serious treatment adverse events since TBI treatment is given with potentially hepatotoxic drugs [24]. While none of the regimens currently recommended by various international guidelines show superiority in terms of efficacy [89], differences in treatment adherence could be significant, particularly in the treatment of migrants with TBI. Indeed, only 69% start the treatment among migrants who tested positive for TBI and, among those who initiated treatment, 74% successfully completed the prescribed regimen, reflecting an inadequate adherence level which led to a suboptimal reduction of the burden of TBI in migrant populations [72, 82]. Several factors may contribute to the challenges in TBI treatment among these individuals. The legal status and patient mistrust create barriers to accessing and adhering to TBI treatment, reducing its effectiveness [72]. Additionally, language and cultural barriers impede clear communication and understanding of treatment regimens, leading to poorer treatment outcomes [90].

Isoniazid was the first drug demonstrated to be effective in TBI to prevent progression to active TB and a six- (6 H) or nine-months (9 H) isoniazid regimen has been used for many years with a good safety profile, making it a trusted option for TBI treatment [41]. However, concerns about liver toxicity and drug resistance due to inadequate adherence to long treatment regimens have led to recommendations for shorter, rifamycin-based courses [31]. Indeed, a key point is that isoniazid resistance is the most commonly detected resistance to first-line TB drugs, occurring approximately twice as often as rifampicin resistance in both low- and high-TB burden countries [5, 91–93]. In this context, it can be argued that there should be greater concern about the potential for treatment failure. Therefore, rifamycin-based regimens are often considered more effective than isoniazid monotherapy [94]. WHO, while acknowledging the effectiveness of shorter treatment regimens and their improved adherence profile, continues to prefer the 6 H regimen as it has been extensively studied and demonstrated efficacy, simplicity, low risk of severe side effects, cost-effectiveness, and ease of monitoring, making it the most practical and widely applicable treatment for TBI [55]. While the other regimens rifamycin-based are alternatives and could offer valuable options, particularly the combination isoniazid-rifampicin once daily for three months (3 h), for children under 15 years of age [55].

CDC recommend a rifamycin-based regimen over isoniazid monotherapy. Rifamycin-based regimens are favored due to their efficacy, favorable treatment completion rates, and relatively low hepatotoxicity rates [95]. Moreover, the shorter duration of rifamycin-based regimens, such as the 3 h and the three-month regimen of weekly rifapentine plus isoniazid (3HP), have demonstrated higher treatment completion rates, addressing a common challenge with isoniazid monotherapy [72, 96, 97]. In particular, 3HP is highly recommended for adults and children over 2 years, including those with HIV, as it has shown similar effectiveness to the 9 H regimen, with fewer adverse effects and better treatment completion rates although it may have a higher cost and requires directly observed therapy (DOT) [95]. Indeed, in an open-label, randomized, noninferiority trial, the 3HP regimen administered once-weekly via DOT was found to be as effective as the self-administered therapy (SAT) with 9 H, with the combination therapy group exhibiting half the rate of TB disease [98]. Although a higher proportion of subjects in the combination-therapy group discontinued treatment due to adverse events, the overall incidence of severe toxicities, as defined by the Common Toxicity Criteria [99], was similar between both groups. Any adverse events, including grade 1–2 and serious events, were less frequent in the combination-therapy group than in the isoniazid-only group. There were no significant differences in grade 3–4 events or mortality, and none of the deaths were related to the study drug. Moreover, the combination regimen also demonstrated higher treatment completion rates [98].

CTS states that the 3 h regimen shows no significant improvement in achieving treatment outcomes, including the prevention of disease recurrence or reduction of adverse effects. Furthermore, patient adherence and completion rates, which are critical factors in the success of TB preventive treatment, do not show marked improvement with the 3 h regimen over the established mono-isoniazid alternatives [100]. Consequently, the existing mono-isoniazid regimens remain the preferred second-line treatments, given their proven track record and comparable performance metrics in the Canadian healthcare context [100]. Additionally, it shares similar risks of drug-drug interactions with 4R and 3HP. Therefore, the CTS does not recommended 3 h as a first choice for TBI treatment [100]. Indeed, limited data exist regarding the use of the 3 h regimen in patients without HIV infection [101]. Furthermore, there is a notable absence of studies investigating the efficacy and safety of this treatment approach in migrant populations. In contrast to this recommendation, a retrospective cohort study compared treatment completion rates between 3HP-SAT and 4R for TBI in adults and showed that 3HP had higher treatment completion rates compared to 4R, especially in those aged 50 and older (87% vs. 64%). Additionally, 3HP-SAT was associated with a lower risk of adverse events in individuals aged 50 and older [102]. Another recent South Korean study including 220,483 individuals diagnosed with TBI, compared 9 H, 4R, and 3 h treatments for TBI and found that shorter treatment durations were associated with a higher likelihood of adherence. The non-adherence rates were 36% for 9 H, 22% for 4R, and 18% for 3HP [103]. Although there is a lack of randomized trials in migrant populations, given that adherence to therapy is particularly challenging in migrant communities, it is reasonable to assume that 3HP effects could also be os use among migrants [97]. Importantly, despite the proven effectiveness of the 3HP regimen [98, 102, 103], it is generally unavailable in the European Union, although it is licensed for use in some countries, such as Sweden, under authorization from the national Medical Products Agency [104]. Neither NICE nor NTAC guidelines recommend the 3HP regimen, as rifapentine is not licensed in the United Kingdom or Australia [24, 65]. Both guidelines consider age a critical element in the choice of treating TBI, with people aged less than 35 years having the best balance between toxicity and benefit [24, 65]. Moreover, for individuals under the age of 35, treatment is typically initiated without the requirement of additional risk factors. This decision is based on the assumption that the benefits of treatment outweigh the potential risks and toxicities in younger individuals, as they may have a longer life expectancy and a higher likelihood of developing active TB disease if left untreated [105]. However, while NTAC adopts a more traditional approach, preferring 6 H over other regimens due to its proven effectiveness, long clinical history, availability, and cost [65], NICE recommends the 3 h regimen as the preferred option. According to NICE, 6 H should be considered an alternative if drug interactions are a concern [24]. A controlled, randomized clinical trial conducted in Barcelona, which focused on migrant populations, supports this recommendation, showing notably higher adherence rates in the 3 h arm compared to the 6 H arm. Importantly, no significant differences in hepatotoxicity or side effects were observed between the two study arms [97].

ECDC proposes that TBI can be treated effectively with 6–9 H, 3HP, 3R or 3 h. However, despite these regimens showing the same effectiveness, shorter regimens are preferred to better adherence [41]. In a randomized, noninferiority trial, the 3HP regimen given via DOT was as effective as 9 H-SAT in preventing TB, with a higher treatment-completion rate [98]. However, while DOT has been shown to improve adherence, it may also be perceived negatively by some patients and could potentially serve as limitations for the implementation of the 3HP regimen [100]. Indeed, some individuals may find it condescending and uncomfortable with the idea of exposing their health status to others. This perception of DOT could potentially contribute to TB-related stigma, particularly in migrant communities with limited understanding of TBI [41]. It was suggested that 3HP-SAT regimen with monthly monitoring could be an acceptable strategy for treating TBI, especially when DOT is not feasible [106]. In such cases, Video-observed therapy (VOT) has proven to be a more effective approach to TB treatment observation than DOT and is likely to be preferable for many patients across diverse settings. It offers a more acceptable, efficient, and cost-effective option for supervising daily or multiple daily doses while overcoming logistical barriers [107]. Its effectiveness, however, depends on access to technology and reliable internet connectivity, which may be limited in low- and middle-income countries, and implementation requires initial investment in infrastructure and staff training [108].There remains a significant gap in the evidence regarding this issue, particularly with regard to studies focusing on migrants.

In summary, while isoniazid-based regimens have a long history of proven effectiveness, shorter rifamycin-based regimens, such as 3 h and 3HP and even 1HP, are gaining attention due to their improved adherence rates and comparable efficacy. Shorter regimens like 3HP have shown higher treatment completion rates, especially in older populations, and lower rates of adverse events. However, concerns about drug resistance and hepatotoxicity remain, particularly with long-term use of isoniazid. Evidence from various studies, including those in migrant communities, suggests that shorter, rifamycin-based regimens could be more effective in ensuring better adherence and completion of treatment. Nonetheless, the lack of randomized trials in migrant populations and concerns about the stigma associated with DOT still pose challenges. Further research is needed to optimize TBI treatment, especially for vulnerable groups such as migrant populations from high TB burden countries. Table 3 highlights the similarities and differences in the preferred treatment stretegies for TBI as recommended by key international health organizations.

**Table 3:**
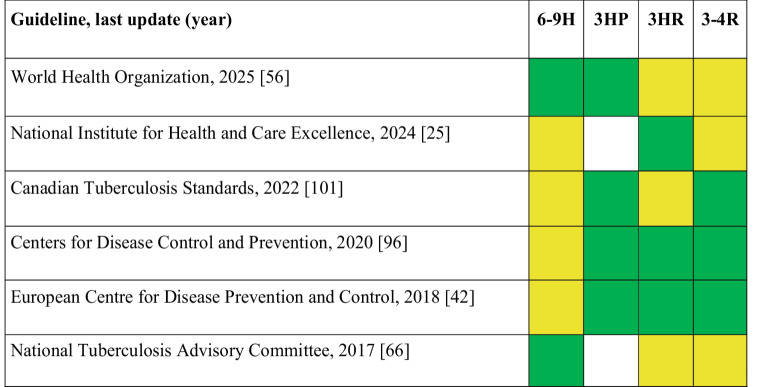
Preferred regimens for tuberculosis infection (TBI) by international guidelines among migrants from high-incidence tuberculosis (TB) countries. For each guideline, preferred regimens are marked in green, those considered alternative in yellow, and white indicates regimens that are not available. 6–9 H: six- to nine-month regimen of daily Isoniazid; 3HP: three-month regimen of weekly Isoniazid and rifapentine; 3 h: three-month regimen of weekly Isoniazid and rifampin. 3-4R: three to four-month regimen of daily rifampin

### Multidrug-resistant or extensively drug-resistant tuberculosis infection treatment

Although specific recommendations may vary based on regional epidemiology and healthcare resources, regimens such as the 3HP and 6 H remained viable options for individuals with suspected drug resistance, provided multidrug-resistant TB (MDR-TB) MDR-TB or extensively drug-resistant TB (XDR-TB) was not present [24]. Indeed, the management of contacts of patients with MDR-TB or XDR-TB with TBI is a complex issue, with limited scientific evidence regarding the efficacy and safety of preventive therapy. Treatment or observation options must be based on a careful assessment of individual risks and the availability of effective drugs. In migrants, however, it is not always possible to trace the index case, which further complicates management. One key issue is that migrants often move across regions or countries, making it difficult to trace the transmission path of the disease and identify the index case (i.e., the initial patient in the outbreak), which is a crucial element in controlling the spread of TB [109]. Additionally, many migrants may lack proper health documentation, or may be outside of formal healthcare systems, which complicates the identification of prior TB cases and their contacts. Without medical records, it becomes challenging to assess their TB history or determine if they are at risk of developing active TB or TBI [110]. Furthermore, language, cultural, and geographical barriers often prevent migrants from accessing healthcare services. These barriers can delay diagnosis and hinder effective contact tracing, as well as treatment initiation [21].

According to ECDC, due to insufficient evidence, TBI treatment is not universally recommended for all contacts of MDR-TB and XDR-TB patients. Instead, it is advised to provide close clinical monitoring, along with guidance and education from an experienced healthcare worker [41]. However, there are promising results in childhood contacts with less than 5 years of age, who are intrinsically a high-risk group for developing TB [111]. A study evaluated the management of children in contact with adult MDR-TB cases, focusing on the effectiveness of treatment for TBI and long-term outcomes [112]. This study observed that 29 (23%) children developed TB, with 90% diagnosed within 12 months. By 30 months, 78% had TBI, with 95% of infections occurring in the first year. TBI treatment was administered to 41 children, while 64 did not receive it. The children who received treatment were younger, had more frequent exposure to smear-positive cases, and showed a higher rate of infection but had significantly fewer cases of TB disease compared to those who did not receive prophylaxis. The study found that 20% of children who did not receive treatment for TBI developed disease, while only 5% of those who received it did. Interestingly, different drug regimen for treatment were reported. The combination of isoniazid, pyrazinamide, and ethionamide was most used with good results. The optimal duration of TBI for MDR-TB contacts remains uncertain although the study suggests that 6 months of directly observed TBI treatment may be more appropriate than longer, unsupervised regimens, especially in areas with high TB burden and limited resources [112].

However, in complex cases such as MDR-TB and XDR-TB, the management of TBI therapy must be highly personalized. The therapy for contacts of MDR-TB patients should be tailored based on the result of drug susceptibility testing performed on the bacteria isolated from the reference patient [113]. In cases where it is deemed appropriate to start treatment for TBI, and NICE guidelines emphasize the role of quinolones. The treatment duration may range from six months for patients at intermediate risk to 12 months for those at high risk (e.g., immunocompromised individuals, young children) [24]. In contrast, due to limited evidence but the significant risk of developing MDR-TB, WHO recommends that the management of MDR-TB contacts be guided by a comprehensive individual risk assessment that carefully weighs potential benefits against harms. In such cases, close clinical monitoring for at least two years is preferred over routine TBI treatment [55]. Two clinical trial recently evaluated safety and effectiveness of levofloxacin as treatment for TBI in adolescent/adults [114] and children [115]. In both trials, the levofloxacin group showed a lower but not statistically significant incidence of tuberculosis [114, 115]. A meta-analysis combining the results of these studies showed a 60% relative reduction in active TB incidence among people receiving levofloxacin compared to placebo. However, the treatment was also associated with an increased risk of adverse events, particularly musculoskeletal issues [116]. If resistance to quinolones is confirmed in the strain isolated from the index case, or if the quinolone is not tolerated, the use of delamanid or bedaquiline may be considered, but only for use in highly specialized settings [24].

In conclusion, TBI treatment for MDR-TB or XDR-TB contacts, particularly for high-risk patients, should be prioritized. The ideal duration of treatment remains unclear, but 6–12 months may be sufficient, and regular follow-up without treatment for TBI is an alternative in the absence of better data. Currently, there is insufficient evidence to definitively support or reject the use of TBI therapy for contacts of MDR-TB and XDR-TB [55, 114–116]. Further research, including controlled clinical trials, is needed to determine the efficacy of preventive therapy in these cases. In the meantime, the best approach may vary depending on available resources and the specific risk factors of each contact.

## Current methodological limitations in the treatment and monitoring of TB in migrants

A critical gap hinders equitable healthcare for migrants, despite policies aimed at improving access [117]. Western medical knowledge, largely derived from Caucasian populations, is not readily generalizable to diverse migrant groups. Applying Caucasian-based laboratory reference intervals and diagnostic algorithms to other ethnicities introduces bias, given known variations in test results [118]. Furthermore, underrepresentation in clinical trials limits understanding of disease presentation, drug metabolism, and treatment efficacy in non-Caucasian populations, especially concerning ethnic variations in drug responses [119, 120]. Finally, epidemiological studies and healthcare policies often overlook the heterogeneity of migrant populations, neglecting variations in genetic predispositions, microbiomes, and prior health risk exposure. Addressing these biases requires a shift towards inclusive medical knowledge, encompassing methodological reassessment, increased research diversity, and culturally sensitive healthcare practices also in TB management [117].

## Conclusions

Ensuring equitable access to TB prevention and treatment services is critical for migrant populations, who may face barriers such as language barriers, legal status concerns and limited healthcare infrastructure in host countries that address the specific needs of migrants, including language-appropriate information, interpretation services, and outreach initiatives targeting migrant communities.

Consequently, healthcare systems in low TB incidence countries must adapt to accommodate the diverse needs of migrant populations while bolstering efforts to maintain TB surveillance, diagnosis, and treatment services for both migrants and residents. This demands collaborative initiatives between governments, non-governmental organizations, and international agencies to develop inclusive strategies that prioritize equitable access to healthcare services and address the intersecting challenges of TB and migration.

## Data Availability

No datasets were generated or analysed during the current study.
